# Biosensing with Quantum Dots: A Microfluidic Approach

**DOI:** 10.3390/s111009732

**Published:** 2011-10-18

**Authors:** Charles H. Vannoy, Anthony J. Tavares, M. Omair Noor, Uvaraj Uddayasankar, Ulrich J. Krull

**Affiliations:** Chemical Sensors Group, Department of Chemical and Physical Sciences, University of Toronto Mississauga, 3359 Mississauga Rd. North, Mississauga, Ontario L5L 1C6, Canada; E-Mails: c.vannoy@utoronto.ca (C.H.V.); anthony.tavares@utoronto.ca (A.J.T.); omair.noor@utoronto.ca (M.O.N.); uvaraj.uddayasankar@utoronto.ca (U.U.)

**Keywords:** biosensor, quantum dots, microfluidics, fluorescence resonance energy transfer, immobilization, nucleic acids, multiplexing, diagnostics, biomarkers

## Abstract

Semiconductor quantum dots (QDs) have served as the basis for signal development in a variety of biosensing technologies and in applications using bioprobes. The use of QDs as physical platforms to develop biosensors and bioprobes has attracted considerable interest. This is largely due to the unique optical properties of QDs that make them excellent choices as donors in fluorescence resonance energy transfer (FRET) and well suited for optical multiplexing. The large majority of QD-based bioprobe and biosensing technologies that have been described operate in bulk solution environments, where selective binding events at the surface of QDs are often associated with relatively long periods to reach a steady-state signal. An alternative approach to the design of biosensor architectures may be provided by a microfluidic system (MFS). A MFS is able to integrate chemical and biological processes into a single platform and allows for manipulation of flow conditions to achieve, by sample transport and mixing, reaction rates that are not entirely diffusion controlled. Integrating assays in a MFS provides numerous additional advantages, which include the use of very small amounts of reagents and samples, possible sample processing before detection, ultra-high sensitivity, high throughput, short analysis time, and *in situ* monitoring. Herein, a comprehensive review is provided that addresses the key concepts and applications of QD-based microfluidic biosensors with an added emphasis on how this combination of technologies provides for innovations in bioassay designs. Examples from the literature are used to highlight the many advantages of biosensing in a MFS and illustrate the versatility that such a platform offers in the design strategy.

## 1. Introduction

The engineering of nanomaterials for use in selective sensing has emerged as one of the most powerful tools for the development of assays based on optical detection. More specifically, the development of fluorescent materials such as semiconductor quantum dots (QDs) has stimulated introduction of a variety of bioassay technologies that offer significant advantages over those that have previously relied on conventional organic fluorophores. The use of QDs as the basis for a biosensor—a tool for the reversible detection and/or quantification of an analyte using biomolecular specificity in combination with a physiochemical transducer—has attracted considerable interest due to the unique optical properties of such colloidal semiconductor nanoparticles, e.g., high quantum yields, broad absorption spectra, narrow and symmetric size-tunable photoluminescent emission spectra, relatively long fluorescent lifetimes, and exceptional photostability with a strong resistance to photobleaching [1–4]. The optoelectronic characteristics of QDs arise through the systematic transformation in the density distribution of the electronic energy levels as a function of the size of the QD, a phenomenon known as the quantum confinement effect [5]. As a result, the nature of the QD surface plays a key role in the optical behavior of the QDs. Thus, the ability to modify the surface of QDs with biomolecules or other moieties through various conjugation methods underpins their versatility.

QD-based biosensors have been incorporated into various applications, often focusing on use of the distance dependence of fluorescence resonance energy transfer (FRET) [6,7], and multiple colors for multiplexed bioanalysis [8,9], for applications such as immunoassays [10,11], nucleic acid detection [12,13], clinical/diagnostic assays [14–16], and cellular labeling and analysis [17,18]. Most reports of such QD-based biosensing technologies are reliant on diffusion-limited solid/liquid interface reactions as typically found in bulk solution. Various types of assays have been demonstrated in bulk solution and on solid substrates such as microbeads and optical fibers [13,19]. Solution-based assays using suitably modified QDs can be challenging because of the need to maintain colloidal stability, which can limit the type and number of biomolecules that can be conjugated to a single QD. Immobilization of such modified QDs at an interface can alleviate this requirement and offers greater versatility in conjugation strategies. However, solid-phase assays often require a significant amount of reagent for immobilization (similar to that used for solution-based assays), and typically suffer from reduced reaction kinetics due to limitations imposed by reliance on interfacial diffusion. Thus, a more competitive technology can be obtained by implementing an assay within a microfluidic system (MFS), where extremely small (*i.e.*, 10^−9^ to 10^−18^ liters) quantities of fluid are transported through microfabricated channels [20]. MFSs can be used to build surface structures on channel walls through chemical reactions, and such modified surfaces can then subsequently be used to interrogate biological recognition. QD bioprobes can be immobilized on channel walls using microfluidic flow conditions, and this same platform then allows for the introduction, manipulation, and handling of multiple analytical samples in sequence, and opportunity for further removal and replacement of QD-based sensing components [21]. Microfluidic technologies offer a number of useful advantages that include: the use of extremely small amounts of reagents and samples; ultra-high sensitivity; high throughput; possible sample processing before detection; short analysis time; *in situ* monitoring; and low cost [20–22]. The large surface area-to-volume ratio and mass transport by non-diffusive means offers the potential for transduction of analytes within seconds to minutes. Microfluidics offers a robust platform and excellent portability, making such assays suitable for point-of-care (POC) diagnostics.

In this review, the convergence of nano- and microtechnologies (e.g., QDs and MFSs) are considered and examples from the literature are introduced to illustrate how mounting assays within a MFS can develop and/or improve biosensing performance. This review will primarily focus on two perspectives: (1) the construction of QD-bioprobes by means of MFS technologies (*i.e.*, synthesis of QDs, immobilization of QDs, and derivatization of QDs *in situ*); and (2) the implementation of a MFS to build rapid clinical/diagnostic technologies (*i.e.*, the use of immobilized QDs for target nucleic acid detection, multiplexed detection of biomarkers, and manipulation of cells for exposure to QDs). For the purposes of this review, the design process centralizes around QDs acting as an integrated sensing component in the MFS, concomitantly having an active part in transduction and serving as a multifunctional nanoscaffold for biorecognition [23,24]. Some examples of assays where QDs serve only as a label are also introduced and represent the simplest integration of QDs for biosensor and bioassay design.

## 2. Microfluidic Systems for Biosensing

Microfluidics can be defined as an area of science and technology involving systems based on microfabricated structures with the capability to precisely control and manipulate fluids constrained to a small scale [20]. As a technology, microfluidics is a multidisciplinary field incorporating chemistry, engineering, physics, molecular biology, and microelectronics. Microfluidics has gained significant attention within the scientific community because it offers many physical advantages, which are specific to their application, when compared to the conventional macro- and meso-scale systems that are in common use. A major driving force behind the rapid development of MFSs is the inherent potential of this technology to be used in biological applications such as biosensing, a topic that has been thoroughly reviewed elsewhere [22,25–27]. MFSs are primed to be a powerful tool for biosensing providing the basis for detection and analysis of biomolecules in a simple yet versatile manner [22].

MFSs normally consist of microscale fluid components and regulators (e.g., pumps, valves, and mixers) integrated into a lab-on-a-chip platform that typically includes a component for signal transduction. The integration capabilities offered by microfluidics allow for the possibility of an extended range of individual laboratory protocols, which include the handling of sample materials, chemical reaction, separation, and detection, to be incorporated and automated within the MFS [25]. With all of these components set in place, a complete MFS functions to greatly enhance the analytical performance of the chemical process from start to finish. A more in depth look at how these components as a whole can be used in each biosensing application will be addressed throughout the review. To further examine the intricacies of microfluidics, the scope of this technology is subdivided into three paradigms pertaining to the specific platform used to carry out the manipulation of fluids at the microscale level. These three paradigms are as follows: (1) continuous-flow; (2) droplet-based; and (3) digital microfluidics. A comparison of these methods is summarized in Table 1.

## 3. Synthesis of Quantum Dots and Subsequent Functionalization

Semiconductor QDs exhibit a multitude of size-, shape-, and structure-dependent chemical and physical properties. Maintenance of the reproducibility of desirable characteristics requires that the synthesis of QDs be conducted in a quality-assured system that enables both kinetic and thermodynamic control of reaction conditions [45]. Conventional methods for the synthesis of QDs rely on high-temperature solution-based reactions derived from organometallic and chalcogenide precursors, which are capped with hydrophobic organic ligands [1,46–48]; however, these synthetic methods are typically complex and often prove difficult in obtaining reproducible high-quality QDs of well-defined size and shape. To address this issue, various research groups have shifted towards the use of a microfluidic approach. This technique has been successfully applied to the synthesis of multiple QD chemistries—CdS [49,50], CdSe [51–53], InP [54,55], along with more complex core/shell structures such as CdSe/ZnS [56,57]. In this section, we discuss the recent developments of incorporating microfluidic technologies into the synthesis of QDs and modification thereof, while addressing both the advantages and challenges that remain in forming bare or functionalized nanoparticles within well-defined microfluidic channels. The interested reader is referred to other reviews for a complete summary about the microfluidic synthesis of QDs [45,58]. Throughout this review, the notation QD*_x_* is used to indicate the QD photoluminescence (PL) peak position at *x* nm. Moreover, the reader should assume that the QD is composed of a CdSe/ZnS (core/shell) material unless otherwise stated.

**Figure 1.**
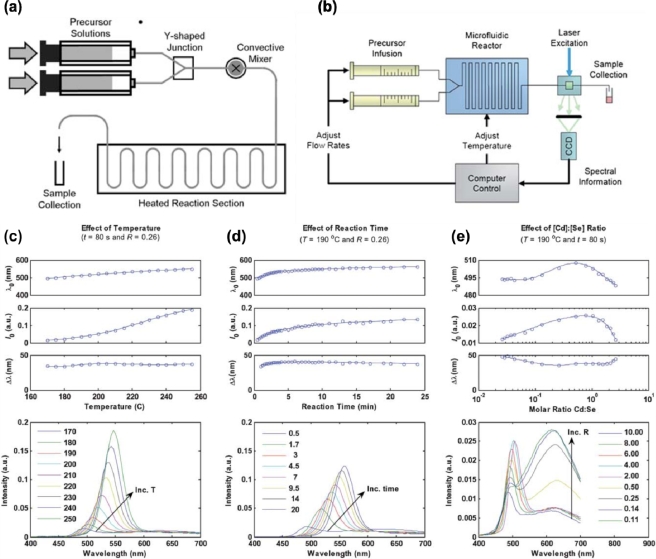
Schematic illustration of typical (**a**) capillary-based; and (**b**) automated chip-based microreactors used for QD synthesis; (**c**–**e**) Graphical representation of the emission characteristics obtained from QD synthesis within a microfluidic reactor with regards to (**c)** temperature; (**d**) reaction time; and (**e**) Cd to Se ratio (for each case, the other two reaction parameters are held constant). Reproduced with permission of the Royal Society of Chemistry [58,62].

The majority of continuous-flow microreactors that are used in the synthesis of QDs are divided into two general types of systems: (1) capillary-based; and (2) chip-based (Figure 1). The capillary-based system represents a simpler method of microfluidic QD synthesis; a set-up requiring only a single length of narrow tubing partially immersed in a heated oil-bath with fluid flow driven by pressure. Glass and polytetrafluoroethylene (PTFE), both of which are chemically inert and acclimated for high temperature procedures, are the materials generally used for the capillary-based system. The second type of system uses a solid platform, otherwise known as a “chip”, which contains the microfluidic channels internally. These chips can be fabricated from a number of materials, which include glass, plastic, silicon, and other polymers. One polymer in particular—poly(dimethylsiloxane), or PDMS—has become an extremely popular choice for much of the exploratory research done in microfluidics [59,60]. PDMS chips are more commonly used for low temperature synthesis, while glass or silicon chips are used for the high temperature reactions due to their chemical and thermal durability. Whatever the choice, both capillary- and chip-based MFSs have been able to offer similar levels of control of QD properties throughout the synthetic process.

The study by Edel *et al.* was one of the first publications that described a synthetic procedure for the synthesis of CdS QDs using a continuous-flow MFS [49]. The system was based on distributed mixing and demonstrated an improvement in the monodispersity of the QDs that were produced. Thus, a combination of miniaturization of the reaction vessel and efficient mixing was able to demonstrate the superiority over traditional bulk scale procedures. Sounart *et al.* then reported the first spatially resolved investigation of QD growth during the synthetic process within a MFS [61]. The results obtained from synthesizing cysteine-capped CdS QDs suggest a diffusion-limited case based on the homogeneity of the reaction and particle nucleation.

However, it was established that the reaction was not entirely diffusion-limited and that it exhibited a slower, rate-limiting final activation process. Krishnadasan and co-workers [59] were able to utilize an automated MFS to systematically control three independent variables—reaction temperature, time, and Cd to Se ratio (Figure 1). This research highlights the versatility of use of a MFS; providing efficient collection of kinetic and thermodynamic data while concurrently optimizing the desired physical properties of CdSe QDs.

While the majority of QD syntheses using a MFS have made use of group II–VI materials, Nightingale and de Mello [54] introduced the synthesis of InP—group III–V materials. Such QDs present a greater synthetic challenge due to the increased covalency of the constituent atoms, which makes it harder to formulate precursor materials and often leads to the production of QDs with obdurate crystal defects [54,63]. The research team was able to accomplish the synthesis in both chip- and capillary-based MFSs by using a reaction of two precursor solutions or a combination thereof, respectively. The first precursor contained InCl_3_, oleic acid, zinc undecylenate—a surface passivation agent, and oleylamine in octadecene—a non-coordinating solvent. The second precursor contained tris-(trimethylsilyl)phosphine in octadecene. As a result, a process that was once deemed to be a significant synthetic challenge became feasible by using a microfluidic approach. More recently, Baek *et al.* were able to accomplish a similar synthesis of InP QDs using a three-stage high-temperature and high-pressure MFS consisting of mixing, aging, and sequential injection stages [55]. The main objective of the researchers was to investigate the role of free fatty acids (*i.e.*, myristic acid) on nanoparticle size. The concentration of free myristic acid was determined to be a major contributing factor of the interparticle ripening processes, providing InP QDs with first excitonic absorption peaks ranging from 495 nm (absence of free myristic acid) up to 650 nm, corresponding to InP QDs with a size of *ca.* 2 to 4.3 nm in diameter, respectively.

Further progress that has been made in the use of microfluidic technology includes investigation of the surface functionalization of the QDs. Kikkeri *et al.* used a continuous-flow microreactor to synthesize high-quality carbohydrate (e.g., mannose or galactose) and carboxylic acid (dihydrolipoic acid, DHLA) functionalized CdSe- and CdTe/ZnS QDs with variable emission peaks in the range of 480–598 nm [64]. In this particular case, the MFS was not only used to synthesize the QDs, but also to modify the surface with biologically relevant moieties. The full capability of MFSs to functionalize QDs has not yet been thoroughly addressed and still requires further investigation.

It should be clear from the preceding examples that the use of a MFS offers many potential advantages in the chemical synthesis of QDs due in large part to the precise control and manipulation of the reaction conditions, high throughput, and a safer working environment throughout the synthesis process [45,64]. One of the attractive features of the MFS approach is the large surface-to-volume ratio provided by the microfluidic channels, which allows for accurate temperature control along with efficient mixing of the precursor solutions [64]. The ability to achieve large-scale production of QDs becomes possible by parallelization, where numerous microreactors operate in unison, or by implementation of a continuous-flow reaction protocol. Continuous-flow MFSs inherently improve reproducibility of synthesis and facilitate *in situ* monitoring [52,53]. Overall, the use of microfluidics changes the entire approach to QD synthesis, potentially creating a new standard for quality of QD technology.

## 4. Quantum Dot-Mediated Fluorescence Resonance Energy Transfer

Fluorescence continues to be one of the most common methods of detection or quantification for various biomolecules [2,65]. More specifically, FRET—the non-radiative energy transfer between an excited state fluorophore (donor) and another fluorophore/chromophore (acceptor) through long-range dipole-dipole coupling—has become a common detection scheme used in bioanalytical methods to monitor binding interactions as well as conformational changes [66,67]. The utility of FRET is unique in generating fluorescence signals sensitive to molecular conformation, association, and separation in the 1–10 nm range, with the FRET efficiency strongly dependent on the distance between the donor and the acceptor. FRET-based transduction using QDs as donors has become a rather popular approach for assay development, in part because enhancements in the FRET efficiency can be achieved as a result of the introduction of multiple pathways for energy transfer. A transduction strategy can be arranged using immobilized QDs and dyes where many QDs have the opportunity to interact with a single acceptor dye, or multiple dyes can interact with a single QD. The enhancement is similar to that displayed in solution with respect to multiple acceptor valency and is described by Equation (1):

(1) $$E=\frac{{\mathrm{nR}}_{o}^{6}}{r^{6}+{\mathrm{nR}}_{o}^{6}}$$

where *E* represents the energy transfer efficiency, *n* is the number of proximal acceptors, *R_o_* is the characteristic Förster distance for the FRET pair at which energy transfer is 50% efficient, and *r* is the distance between the donor and acceptor.

A detailed description of the advantages of using QDs as donors in FRET for solid-phase assays can be found elsewhere [24]. QDs have become increasingly integrated into FRET-based assays, in particular as donors, offering a number of advantages, most of which have been explored in a number of recent reviews [6,7,24]. In counterpoint, QDs have been shown to be less efficient acceptors when used in combination with molecular dye donors in FRET studies [68,69]. QDs have the potential to serve as excellent acceptors in chemiluminescence resonance energy transfer (CRET) where no excitation source is used [24]. Reasons for this behavior stem from the fact that QDs possess higher quantum yields than molecular dyes and have a higher cross-section for the absorption of light. They also tend to be more resistant towards photobleaching, allowing for application in long-term assays. Another benefit of QDs is their broad absorption spectrum in the UV region, which can be used advantageously to avoid the direct excitation of acceptor dyes. Transduction via FRET also enables multiple methods for data analyses. Ratiometric detection is often used and easily accounts for differences in assay preparation amongst multiple analyses and instrumental drift. The narrow symmetric emission profile of QD PL facilitates deconvolution of acceptor signals for recovery of absolute signals [70]. This section serves to address the various aspects of FRET that are significant to QD-based biosensors and focuses on the integration of QD mediated FRET transduction in MFSs.

**Figure 2.**
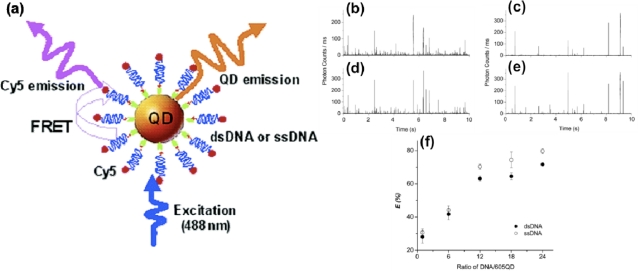
(**a**) Schematic illustration of a single QD-based DNA biosensor; (**b**–**e**) Representative traces of fluorescence bursts from (**b**,**d**) QD_605_/dsDNA/Cy5; and (**c**,**e**) QD_605_/ssDNA/Cy5 complexes in a capillary flow; (**b**,**c**) show the Cy5 bursts in the acceptor channel; (**d**,**e**) show the QD_605_ bursts in the donor channel; (**f**) The change of FRET efficiency with the ratio of DNA-to-QD_605_. (ds = double-stranded, ss = single-stranded) Reproduced with permission of the American Chemical Society [73].

Microfluidics has been known to facilitate single molecule detection (SMD) studies, and the use of SMD for QD mediated FRET has also been demonstrated [71]. SMD studies are useful to determine patterns and distributions that may otherwise be masked by ensemble averages [72]. SMD studies are usually performed by monitoring a fixed volume of solution, more commonly known as the detection volume. Traditionally, analytes are allowed to diffuse into this fixed volume in order to be detected. A continuous-flow system as found in capillaries and microfluidics ensures that multiple analytes are moved across the detection volume, increasing the probability of detection. Zhang *et al.* made use of microscale flow in capillaries to facilitate SMD studies of QD mediated FRET (Figure 2) [73]. A QD-DNA bioconjugate was used for these studies and was comprised of 3′-biotinylated nucleic acids immobilized onto a streptavidin-QD_605_. The 5′ end of the DNA was further labeled with a Cy5 dye to serve as a FRET acceptor. A 488 nm laser was focused onto the center of a 50 μm capillary that contained the QD-DNA conjugates. Pressure driven flow was used to drive the sample across the detection volume of the laser. Compared to bulk measurements, improved FRET efficiencies were obtained using the continuous-flow capillary system. Both single- and double-stranded DNA were analyzed in this system. For the double-stranded DNA, the measured distance (calculated from FRET efficiencies) between the donor and acceptor was reduced from 12 nm in bulk solution to 9.6 nm under capillary flow. This reduction in the separation distance was attributed to the deformation of DNA under hydrodynamic flow. This has the potential to provide an important advantage, as low FRET efficiencies are usually observed for double stranded DNA in which the acceptor dye is located on the distal end [74]. The effect of flow rate was also investigated in a later study by the same research group [75]. It was observed that a flow rate of 6 μL/min led to an increase in FRET efficiency, with a FRET efficiency of 44.5% in the flow system as compared to a FRET efficiency of 9.1% in bulk solution conditions. This system was used to develop a sandwich-based nucleic acid assay for a 50-base pair target. No FRET signal was observed in bulk solution experiments; however, in capillary flow a FRET signal was observed. Using the FRET efficiency, the distance between donor and acceptor was calculated to be 12.6 nm under flow, while in a stationary system the distance was theoretically calculated to be 22–24.5 nm.

The ability to conjugate multiple biomolecules onto the surface of a QD is beneficial for studies of SMD. Having more than one biomolecule on the QD surface translates into multiple FRET acceptors being present, which improves the FRET efficiency. This also allows for multiplexed detection as different biomolecules can be immobilized onto a single QD. When considering biosensing, one opportunity made possible by QD-based detection is the potential for multiplexing [4]. Optical multiplexing with QDs allows for concurrent detection of multiple analytes in a single sample without the need for spatial registration. The limitations imposed by organic dyes for such applications are related to their comparatively broad and slightly structured emission bands. QDs provide narrow, symmetric emission spectra with Gaussian-like PL curves, allowing for the simultaneous excitation of multiple fluorescence colors at a single excitation wavelength within a given wavelength range and the straightforward use of deconvolution schemes to quantitatively resolve individual QD fluorescence signals [24,70]. Multiplexed detection capabilities within a MFS was demonstrated by Zhang *et al.* [76], in which two color coincidence detection and FRET was used to analyze two different nucleic acid sequences using a single color of QD. The target strand of one DNA was labeled with Alexa Fluor 647 for FRET-based detection. The other target strand was labeled with Alexa Fluor 488, which could be directly excited by a laser at 488 nm. Monitoring the presence of the PL of Alexa Fluor 488 and the QD in the same detection volume was indicative of a positive signal for the nucleic acid sequence. The ability to immobilize multiple sequences onto a single QD improved the sensitivity of two color coincidence detection as it concentrated multiple fluorophores in a small volume. Also, use of capillary flow allowed for only a small volume of solution to pass through the detection volume. This reduced the coincidence of free labeled nucleic acid with a QD, which would have registered as a positive signal in two color coincidence detection. Additional advantages of operating SMD in capillary flow are that each molecule is excited only once, thereby reducing the incidence of photobleaching, high signal-to-noise ratio, as well as near-zero background noise without interference from background fluorescence. However, this comes at a cost of running increased volumes of solution through the capillary in order to maintain a sufficient signal-to-noise ratio. Puleo *et al.* [77] were able to overcome this issue by using an integrated closed loop, rotary pump, fabricated with the use of multilayer soft lithography. In this design, the microchip consisted of a closed loop, a part of which was interrogated by a laser-induced fluorescence (LIF) microscope. The loop could hold approximately 5 nL of solution, which was driven through the loop using a set of peristaltic pumps controlled by solenoid valves. This allowed the same sample to be analyzed multiple times, resulting in improvement of the sensitivity of detection. It was demonstrated that the sensitivity achieved using this device was similar to that obtained by continuous-flow capillaries, but a much smaller volume was used. The repeated sampling of a single volume of solution could increase the probability of photobleaching, and this was also investigated in the study. It was determined that a continuous monitoring time of 400 s produced results that were similar to continuously injecting the solution through a regular capillary. At longer analysis times, the effect of photobleaching was noticeable.

For microfluidic-based separations, LIF is a common detection scheme because high sensitivity methods are required to measure the small number of molecules in the small volumes of microfluidics. An alternative detection method was developed by Zhao *et al.* [78]. The CRET between luminol and a CdTe QD was used to detect various organic compounds of biological interest in solution as they were separated by capillary electrophoresis; a technique that has become a major application of microfluidics. It was observed that by mixing luminol, mercaptopropionic acid coated QDs, and sodium hypobromite, energy transfer could be observed from the luminol to the QD. This energy transfer was interrupted by the presence of a number of active groups, such as amines, thiols, organic acids, and steroids, in solution. To integrate this system with a MFS, sodium hypobromite was introduced at the end of the separation channel and the running buffer contained luminol and the QDs. The luminescence of the QD via CRET was monitored at the end of the separation channel. The reduction in CRET was used to identify analytes as they eluted from the separation component of the MFS, providing sensitivities that were 10–1,000 times greater than those obtained by LIF and other common detection schemes. This system was further used to demonstrate the analysis of amino acids secreted from a single cell. In this case, a single cell was isolated in a microfluidic channel and was subjected to a series of electrical shocks. The separation was then performed on the molecules released from the cell, and the analytes were detected by monitoring the inhibition of CRET. The analytes detected from the cell were in the attomole/cell range, illustrating the sensitivity of this technique. Another use of capillary electrophoresis was demonstrated by Li *et al.* in which they explored FRET-based immunoassays [79]. Mouse immunoglobulin (IgG) was conjugated to a CdTe QD_532_ that served as the FRET donor, while goat anti-mouse IgG was conjugated to a CdSe/ZnS QD_632_ that served as the acceptor. Upon immunoreaction, FRET was observed between the two QDs, providing FRET efficiencies of *ca.* 70%. While it was possible to directly excite the acceptor, capillary electrophoresis was used to separate the acceptor QDs that were not bound to the donor QD, thus reducing the incidence of false positives. An increase in intensity of the acceptor in the immunocomplex, as compared to that of the free QD was observed and attributed to FRET. Thus, the separation capability of capillary electrophoresis enabled the use of QDs as both the donor and acceptor, providing for a sensitive and versatile analysis method.

Another important property of flow achieved by moving to a microchannel scale is a low Reynolds number. A laminar flow regime is in place for low Reynolds number, and this typically provides for diffusion-limited reactions (in flat planar channels). This property is useful in the study of the kinetics with regards to various processes. Ho *et al.* [80] were able to demonstrate the use of a T-channel microfluidic structure to determine the complexation kinetics of plasmid DNA with chitosan, a cationic polymer with favourable properties for gene delivery. The plasmid DNA was conjugated to streptavidin-QD_605_ bioconjugates via a biotin label, and the chitosan was partially labeled with Cy5 to serve as a FRET acceptor. By introducing these solutions simultaneously through a T channel, complex formation initially occurred only at the physical interface of the two streams of solution. The resulting FRET signal was used to monitor complex formation between the DNA and chitosan. In this system, the temporal resolution of the reaction became spatially resolved along the length of the microchannel. Simplification of kinetic studies was achieved by imaging of the entire channel and providing for real-time monitoring. In addition, the laminar flow regime allowed the diffusion coefficients of the complexes to be determined, which provided information about the size of the complexes as they formed. The results obtained were corroborated by physical measurements made using electron microscopy [81].

## 5. Strategies for Immobilization of Quantum Dots and Quantum Dot-Bioconjugates

Solid-phase assays are becoming an increasingly popular approach in the development of biosensors that integrate QDs. Immobilization of QDs at an interface offers numerous advantages with respect to solution-based analyses. Most importantly, the necessity to maintain colloidal stability, which can often at times impose limitations or influence the performance of an assay, no longer becomes a requirement. This provides the opportunity to conduct analyses in complex matrices of varying pH or ionic strength [24]. One such example occurs in the case of thiol-alkylcarboxylic acid coated QDs. These typically require that the pH of the solution be kept alkaline and low in ionic strength in order to maintain colloidal stability. In contrast, some biorecognition events such as DNA hybridization are more efficient at neutral pH and at higher ionic strength to screen backbone phosphate charge. Interfacial immobilization of QDs permits analyses at solution conditions that are conducive to hybridization without concern for colloidal stability. Immobilization also simplifies washing and/or extraction procedures, facilitating the removal of adsorbed material and increasing stringency conditions. In a microfluidic channel, regeneration and reusability of the sensing surface can be readily achieved if biorecognition is a reversible process. From a practical perspective, it is also the case that immobilization onto solid supports minimizes any loss of QDs into the environment [19]. These advantages enable the development of stable and regenerable QD-based biosensors with improved sensitivity and rapid response.

The interfacial immobilization of QDs for biosensing applications requires consideration of stability, immobilization density, and compatibility for subsequent biomolecule conjugation [24]. The latter is often the most important consideration, largely because the surface of the QD must be activated with ligands that are suitable for both immobilization and possible subsequent ligation. The size and orientation of the biomolecule are further factors that impact on assay performance and are often influenced by the coupling method. Progress that has been made in preparation of QDs with novel surface ligands, as well as availability of coupling chemistries that provide orthogonal functional groups to common biological groups (e.g., –SH, –OH, –CHO, –COOH, –NH_2_), in combination allow for significant flexibility in regards to the methods that can be chosen for immobilization and bioconjugation. Coordinate/dative bonds, physical entrapment, passive adsorption, layer-by-layer assembly (LbL), and bioaffinity-mediated interactions are just some examples of the more commonly used immobilization strategies.

In MFSs, two opportunities exist for immobilization: (1) coupling to the interior channel walls; mainly the glass component, although methods for the derivatization of PDMS have also been reported [82]; and (2) use of pseudo-stationary phases that can be introduced into channels such as polymeric or magnetic microbeads (Table 2).

### 5.1. In-Channel Assays

**Figure 3.**
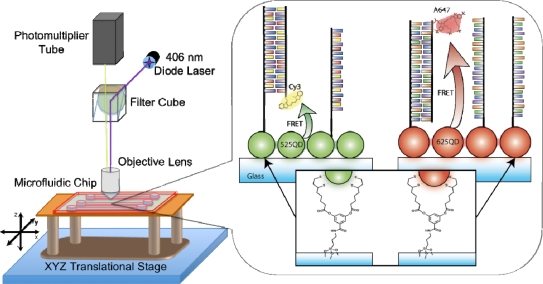
Schematic illustration of the in-house laser scanning epifluorescent microscope used for fluorescence imaging (**left**) and the in-channel assay design with immobilized QD_525_ and QD_625_ conjugated with amine-terminated probe nucleic acids that are subsequently hybridized with Cy3- or Alexa Fluor 647-labeled target oligonucleotides, respectively (**right**).

Interfacial immobilization of QDs has been adopted in many on-chip solid-phase assays for the detection of a wide variety of analytes. Our research group has developed in-channel QD-FRET based assays for the detection of nucleic acids using various immobilization strategies [21,85,87]. For example, streptavidin-coated QD_525_ conjugates were immobilized to a biotin functionalized glass slide. The immobilized QDs were then subsequently conjugated with a 10-fold excess of biotinylated probe oligonucleotides and then subjected to Cy3 labeled complementary and non-complementary targets. Delivery of QDs, probes, and target nucleic acids were accomplished in-channel, with the former being facilitated by electro-osmotic flow (EOF) and the translocation of oligonucleotides by electrophoresis. Hybridization provided the necessary proximity for FRET between the QD and Cy3 labeled oligonucleotides [87]. The assay displayed excellent resistance to non-specific adsorption, which was ascribed to the streptavidin coating on the QDs. The drawback in this design was the limited sensitivity of the assay. This was attributed to the intrinsic size of the streptavidin-QD conjugates, where the protein dimensions (5.6 × 5.0 × 4.0 nm) [94] and a polyethylene glycol (PEG) tether significantly contributed to a large QD-Cy3 separation distance. FRET efficiencies determined by time-resolved fluorescence measurements of less than 20% were reported. In an unrelated design, transduction of nucleic acid hybridization was demonstrated in a reusable format where the sensing surface could be regenerated for multiple cycles of analysis. In this assay, 3-mercaptopropionic acid (MPA) functionalized QD_525_ were concurrently conjugated with two different nucleic acid sequences via self-assembly through a terminal thiol. One sequence served to hybridize with a complementary probe oligonucleotide that was immobilized on the glass slide of a microchannel within a PDMS-glass chip. The other sequence served to transduce hybridization via FRET sensitized emission from Cy3 labeled targets. Immobilized QDs could be removed by duplex destabilization by increasing the applied voltage, resulting in a combination of shear force and Joule heating leading to dehybridization. It was demonstrated that destabilization of hybrids could also be achieved by the addition of formamide into the running buffer. Multiple cycles of regeneration were possible as new QD-oligonucleotide conjugates could be delivered electrophoretically to the areas of the glass channel that were coated with immobilized nucleic acid [21]. The results of the chip-based assay compared favorably to those obtained from previously developed solid-phase assays [19] on optical fibers. Our research group has also used microfluidics to develop a QD-FRET based assay with improved FRET efficiency and enhanced sensitivity. In this work, transduction of nucleic acid hybridization was demonstrated in two separate FRET pairs: (1) QD_625_ paired with Alexa Fluor 647; and (2) QD_525_ paired with Cy3 (Figure 3). Interfacial immobilization of QDs was facilitated by ligand exchange of the MPA coating of the QD with multidentate surface ligands as previously developed [95]. Subsequent conjugation of immobilized QDs with amine terminated nucleic acid probes was accomplished through activation of the remaining MPA surface ligands with N-(3-dimethylaminopropyl)-N'-ethylcarbodiimide (EDC) and *N*-hydroxysulfosuccinimide (NHS). The remaining surface sites of the immobilized QDs were passivated with unlabeled oligonucleotides, which have shown the ability to adsorb strongly onto the surface of MPA functionalized QDs [96]. Hybridization with complementary targets generated FRET sensitized Cy3 or A647 fluorescence with improved FRET efficiencies in comparison to assays that used streptavidin-QDs. Excellent selectivity was also achieved against non-complementary labeled targets [85]. This preliminary work is the prelude for the development of a multiplexed color assay capable of the concurrent detection of two nucleic acid targets. An important advantage of integrating these assays into an electrokinetically controlled MFS is that it allows for rapid in-channel assay assembly with minimal sample reagent consumption. An electrokinetically controlled environment has also been previously reported to enhance the stringency of nucleic acid hybridization, where elevated voltages can shear fully complementary duplexes for assay regeneration [21,97] and can even allow dynamic tuning of stringency to discriminate single nucleotide polymorphisms (SNPs) [98].

A FRET-based assay to monitor protease activity was reported by Crivat *et al.* [83], and in this design, QD immobilization was accomplished through the use of polyelectrolyte multilayer (PEM) deposition, which involves the deposition of alternating layers of positively and negatively charged polymers where the QDs are immobilized within. The microfluidic channels facilitate the introduction of a PEM, utilizing poly(allylamine hydrochloride) and poly(styrene sulfonate) as the positively and negatively charged polymers, respectively, along with a layer of MPA functionalized QDs. A rhodamine labeled peptide, neutrotensin, was immobilized onto the outermost layer of the PEM such that FRET was observed between the immobilized QDs and the rhodamine dye on the peptide. The introduction of a proteolytic enzyme, trypsin, caused the cleavage of the peptide, which subsequently removed the dye from the surface, thereby reducing the FRET signal. Essentially, the activity of the enzyme could be monitored through a decrease in FRET signal. The advantages of using PEMs arise from their potential use in cell-based assays, wherein it has been shown that PEMs are biocompatible and promote the adhesion of cells to surfaces while potentially shielding the cells from any possible harmful effects provided by QDs [83].

**Figure 4.**
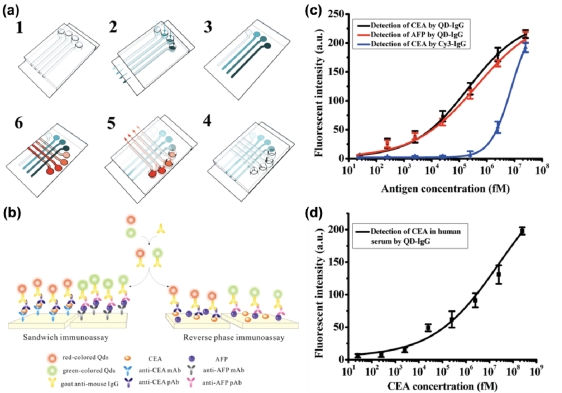
(**a**) Schematic of a multiplexed diagnostic detection system based on microfluidic bio-chip fabrication in a step-wise fashion; (**b**) Schematic of a sandwich and reverse phase immunoassay based on QD-IgG fluorescent probes and microfluidic protein chip. Dose-response calibration curves for the detection of (**c**) CEA and AFP; and (**d**) CEA in human serum utilizing QD-IgG probes and a microfluidic bio-chip. Reproduced with permission of the American Chemical Society [99].

Innovative technology based on multiplexed diagnostic detection has put emphasis on the quantification of various cancer biomarkers and infectious diseases for use in clinical/diagnostic technologies. Hu and co-workers were able to attain some promising results using a versatile microfluidic bio-chip that incorporated both sandwich and reverse phase immunoassays to achieve ultrasensitive and selective detection (Figure 4) [99]. The design of the microfluidic bio-chip introduced fluorescent QDs bioconjugated to a secondary antibody (goat anti-mouse IgG), which enabled rapid detection of carcinoma embryonic antigen (CEA) and α-fetoprotein (AFP). To optimize the system and reduce background signals, the group blocked adsorption by the QD-IgG bioconjugate with 5% BSA, added Tween-20 to washing solutions to minimize non-specific absorption, and stabilized the fluorescence with reduced l-glutathione. The integration of the QD-IgG bioconjugate into the microfluidic bio-chip was ultimately able to improve the LOD of cancer biomarkers to 250 fmol/L in buffer and 2.5 pmol/L CEA in human serum (Figure 4). These results represent a considerable improvement in performance in comparison to conventional organic dyes.

### 5.2. Microsphere Bead Assays

The use of polymeric or magnetic microsphere beads in conjunction with microfluidic devices has become commonplace in assay development. The surfaces of microsphere beads are easily modified for bioconjugation and these substrates can be readily manipulated in MFSs by dielectrophoresis [92]. The large surface area associated with these substrates allows for the coupling of many biomolecules, which can greatly enhance the efficiency of an assay [91]. In these designs, the beads can act as a stationary phase or scaffold for the immobilization of biomolecules. The QD often serves as a label to transduce the biorecognition event. In other strategies, the beads can be encoded with various colors of QDs for multiplexed analysis or can be co-immobilized along with the biomolecules. An advantage of using QDs to encode beads is the ability to excite multiple colors of QDs using a single wavelength [100]. Microbeads also facilitate diffusional mixing in laminar flow regimes, which can greatly enhance the efficiency of an assay. Magnetic beads provide biocompatibility and the ability for ease of isolation by use of an external magnet [91]. This permits routine washing or extraction steps, which can greatly enhance the performance of an assay without the need to be concerned about substrate stability. The following section describes examples of bioassays that integrate polymeric or magnetic beads into the design.

Immunoaffinity is a popular choice for introduction of selectivity in bead-based assays because various chemically modified polymeric beads can be readily coupled with antibodies. It is also possible in some cases to use passive adsorption on polymeric materials to facilitate the immobilization process. Yun *et al.* have developed a QD-bead based immunoassay that uses a MFS capable of single bead isolation and interrogation [92]. Streptavidin-coated, 10 μm polystyrene beads were derivatized with biotinylated protein-A and delivered into a PDMS-glass microfluidic chip. On-chip single bead isolation was facilitated by use of a pneumatically controlled micro-chamber integrated into the PDMS layer. The 50 μm × 50 μm × 30 μm chamber consisted of two gates that were controlled by channels imprinted in and above the PDMS layer. Upon application of vacuum to either channel, each gate could rise and allow a micro-bead to enter the chamber. In the closed position, each gate did not completely seal the channel. A small gap permitted the flow of solution but prevented beads from entering or leaving the chamber. Sequestered beads were then further conjugated with human IgG antibody. Anti-human IgG-QD conjugates were subsequently delivered into the chamber and captured by the IgG conjugated bead. Antibody detection was facilitated by the fluorescence of QD_605_ upon excitation from a 470 nm source. Fluorescence images of isolated beads were captured using a charge-coupled device (CCD) camera. A LOD of 0.1 μg/mL was reported for the antibody [92]. A multiplexed QD-microsphere immunoassay was reported by Lucas *et al.* [93], in which highly carboxylated polystyrene-polyacrylic acid latex beads were coated with amino-PEG QDs via passive adsorption. The authors referred to these configurations as nano-on-micro (NOM) moieties. Specifically, two NOM moieties were designed in this assay: NOM-605 and NOM-655, where 605 and 655 refers to the PL max of the immobilized QDs. NOM-605 and 655 were then conjugated with bovine serum albumin (BSA) and mouse IgG, respectively. Varying concentrations of anti-BSA, anti-mouse IgG or equal mixtures of the two were then introduced and antibody capture resulted in agglutination of the microbeads. Detection was facilitated by coupling the output of a 380 nm light-emitting diode (LED) into the channel via an optical fiber and positioning the detection fiber at a 45° angle relative to the source. Aggregation of the NOM(s) caused an increase in the scatter of source photons, which identified the presence of antibody. The decrease in PL from either QD_605_ or QD_655_ signified whether the antibody was anti-BSA or anti-mouse IgG, respectively. Selective detection of each individual antibody was demonstrated, and a LOD of 100 ng/mL was reported for the scatter response. A LOD of 200 ng/mL was obtained for transduction via decrease in QD PL. The system was also subjected to simultaneous exposure of equimolar amounts of the antibodies in both a two-well plate and on-chip formats. The assay demonstrated multiplexed detection and provided a LOD of the total antibody concentration of 50 ng/mL from light scatter [93]. Aptamer-based recognition for the detection of thrombin was demonstrated using a sandwich assay format with magnetic beads and QDs [91]. The assay was conducted using a novel chip design that used eight reaction wells that were coupled with microchannels to a center exit reservoir. This exit well was connected to a syringe pump to remove solution from the chip after each washing step. Streptavidin functionalized beads and QDs were conjugated to two DNA thrombin-binding aptamers, each recognizing separate epitopes of the protein. The aptamer bioconjugated beads were fixed in each reservoir by external magnets under the chip. Various concentrations of thrombin were added to each reaction reservoir followed by washing steps and then addition of the aptamer coated QDs. Chips were then imaged using fluorescence microscopy, and QD PL was used to transduce thrombin recognition. The dynamic range of the assay was reported to be 100–1,000 ng/mL and the LOD was determined to be 10 ng/mL. The selectivity of the assay was demonstrated using an aptamer that was non-specific for thrombin, a 700-fold excess of human serum albumin (HSA) and 72 μg/mL of prothrombin. In all cases, no appreciable fluorescence was detected above the signal obtained from the controls. The chip-based results were compared to an assay conducted in a 96-well plate, which displayed similar performance. However, the on-chip analysis was considered to have the favorable advantages of automation, increased assay efficiency, and the opportunity to operate in a multiplexed format [91].

Jokerst *et al.* [101] reported the use of a QD-based MFS for the multiplexed detection of various biomarkers for clinical/diagnostic application based on a microsphere bead support. In this example, QDs functioned as detection elements for the multiplexed quantification of three cancer biomarkers: CEA—colon cancer, cancer antigen 125 (CA125)—ovarian cancer, and human epidermal growth factor receptor 2 (Her-2)—breast cancer. The authors were able to exploit the optical properties of QDs to provide useful quantitative cancer biomarker information using both serum and whole saliva clinical samples. The QD-based MFS made use of a typical sandwich immunoassay approach, where agarose beads served as a solid-phase support structure for capture antibody immobilization. The three analytes provided linear dose responses ranging from 0–100 ng/mL, 0–60 ng/mL, and 0–400 U/mL for CEA, Her-2, and CA125, respectively. The LOD for CEA and Her-2 were 0.02 ng/mL and 0.27 ng/mL, respectively, with no LOD given for CA125. More specifically, the system proved to be almost twice as sensitive to that of an ELISA assay and demonstrated the advantages of combining QDs and a MFS to obtain high intensity signals, low LODs, and short assay times.

The use of QD encoded beads integrated into a MFS to perform low volume multiplexed assays was demonstrated by Riegger *et al.* [90] in a duplex sandwich immunoassay. They used two sets of 150 μm polystyrene beads, each set encoded with a single color of QD. The beads were functionalized with the antigen for hepatitis A and tetanus, allowing the antibodies for these infectious diseases to bind to the bead. The presence of antibodies was then transduced by a labeled detection antibody. A centrifugal microfluidic device was used for the various sample handling steps such as reagent delivery and washing steps. An imaging-based detection system was used to facilitate the detection of all beads simultaneously. To ensure that all beads were imaged, the beads were packed into a monolayer using a chamber that had a height slightly larger than the beads. To facilitate portability, a high-power 475 nm LED was used for excitation of all QDs and the assay label, and a color CCD-camera was used for detection. FluoSpheres^®^, which are beads packed with organic dye molecules, were used as the assay label due to the high sensitivity offered. Using an incubation time of only 180 s and washing time of 20 s, LODs of 215 mIU/mL and 158 mIU/mL were obtained for the hepatitis A assay and the tetanus assay, respectively. While the imaging detection module has the advantage of analyzing all beads in parallel, there are limitations to the total number of beads that can be analyzed. Any inhomogeneity in the image would affect the data obtained, as was observed in the experiment due to the fact that the interior of the beads were not uniformly packed with QDs.

**Figure 5.**
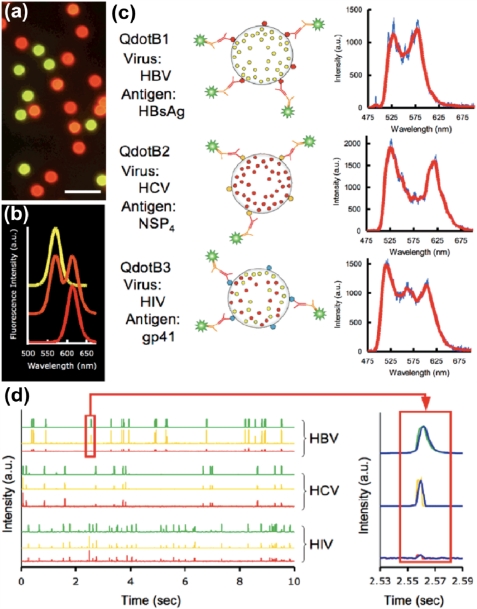
(**a**) Fluorescence image of a collective set of three QD encoded beads; and (**b**) the corresponding QD PL spectra; (**c**) Schematic illustration of the three different QD encoded beads with their corresponding surface conjugated antigens (e.g., HBV, HCV, and HIV) (**left**) and corresponding PL spectra (**right**). Spectra were taken of assayed QD barcodes flowing in a microfluidic channel; (**d**) Photodetector data collected over 10 s intervals for HBV, HCV, and HIV detection sensitivity experiments. Each detector investigated a separate bandwidth determined by emission filters. The peaks from one HBV detection event are enlarged on the right, depicting how detection peaks appear in all three channels. The slight variation in the width and tail of the top (green channel) peak compared with the peaks for the bottom two (yellow and red channels) illustrates different types of solid-state detectors being used within the detection platform. Reproduced with permission of the American Chemical Society [89].

Another common approach for analyzing encoded beads is the analysis of single beads in a flow stream with the use of a flow cytometer. This has the advantage of spectral-based bead detection, which is not affected by the spatial distribution of dye inside the bead. In addition, a larger number of beads may be analyzed and is limited only by the detection instrumentation. Microfluidics can be used instead of a flow cytometer [102], as was demonstrated by Klostranec *et al.* in the use of a MFS for multiplexed assays using QD encoded beads [89]. A triplex immunoassay for infectious diseases (*i.e.*, human immunodeficiency virus (HIV), hepatitis B, and hepatitis C) was performed using three sets of beads that were labeled with varying combinations of two colors of QDs (Figure 5). The antigens were immobilized on the surface of the microbeads and incubated with sample solutions to bind labeled antibodies. Subsequently, the beads were washed and introduced into the microfluidic device. The microfluidic device made use of focusing by electrokinetic flow to ensure that the beads crossed the detection volume in single file allowing for single bead detection. In electrokinetic focusing, the sample stream is sandwiched between two streams known as the sheath flow, with all streams intersecting at 90° to each other. Mixing of the three streams is avoided due to the laminar flow regime of microfluidics [103]. A laser at 488 nm was focused onto a certain spot in the microchannel and was used to excite the QDs as well as the fluorophore conjugated to the detection antibody. The emitted fluorescence was simultaneously detected using a PIN photodiode for green wavelengths, and an avalanche photodiode for the yellow and red bandwidths. Using this detection system, the instrument could analyze 70 beads per minute. Focusing by use of electrokinetic flow allowed the facile control of sample stream width, involving only adjustment of the voltage applied across the different microchannels. In addition, the use of a sheath flow prevented direct contact with channel walls reducing any non-specific interactions. The use of electrokinetic controlled flow rather than pressure driven flow makes the instrumentation more compact, and the complexity of the system is further reduced with the use of QDs. The MFS completed the analysis of three infectious diseases in less than an hour, using less than 100 μL of sample and was found to be 50 times more sensitive than FDA approved methods.

An alternative to incorporating QDs inside a bead is to immobilize the labels onto the surface for purposes of encoding. This offers simplification for bead encoding. The use of this approach for a two-plex immunoassay was demonstrated by Ma *et al.* [88]. Two different colors of QDs were immobilized onto a microbead using the LbL approach, and the ratio of the two colors were used for encoding beads. A PEM consisting of positively charged polymer (poly(allylamine hydrochloride)) and negatively charged polymer (poly(styrene sulfonate)) was immobilized along with the QDs, with different colors of QD occupying different layers. This placed a restriction on the intensity ratios that were possible because lower layers of QDs were found to have a lower intensity once a layer of QDs was immobilized on top. The outermost layer of the bead was used for the immobilization of the capture antibodies. These beads were used for a proof of concept immunoassay to detect human IgG and rabbit IgG. All assay steps were performed in bulk solution with the final analysis being performed in a microfluidics environment.

An LIF system was used for detection, and a 488 nm laser was the single source of excitation of the QDs and the fluorescein isothiocyanate (FITC) conjugated detection antibodies. The scatter of the laser was also used to verify the presence of a bead. The use of microfluidics facilitated single bead analysis using coincident detection applied to four channels of information. One channel used the scatter of light from the beads to register the presence of a bead in the flow stream. Two channels of luminescence from the dots were used to decode the bead. The remaining channel was used to monitor the luminescence intensity of the dye conjugated detection antibody.

The above examples serve to demonstrate the advantages of using microfluidics for bead-based assays that use QDs either as a label for the detection of biomolecules or as an encoding mechanism for solution phase multiplexed assays. The novel chip design strategies incorporated into these assays serve to enhance their analytical performance by providing a method for routine bead manipulation, less intensive extraction and washing steps, and facile automation.

## 6. Cellular Analysis

Cell analysis and processing has greatly benefited from the field of microfluidics, where similarities between the dimensions of cells and microchannels (10–100 μm) are able to facilitate spatial and temporal manipulation of cells and their microenvironment. Flow focusing [104], electroporation [105], dielectrophoresis [106], and cell lysis [107] are just some of the routine procedures that can be conducted rapidly and with excellent precision using microscale phenomena. Flow focusing and cell trapping can be used to interrogate single cells individually [108]. Electric field driven phenomena such as electroporation can be used to actively deliver reagents like DNA or QDs into cells [109], while dielectrophoresis can be used to perform on chip separation of transformed cells from those that remain unaffected. In addition, dielectrophoresis provides the capability of segregating live cells from dead cells [110]. Electrokinetic phenomena can also be used to lyse cells for subsequent analysis of their content and to perform on chip flow cytometry. All of these tasks can be integrated within a single microfluidic chip to develop a micro total analysis system (μTAS) [111,112]. Once again, the increased surface area-to-volume ratio associated with microfluidic channels becomes advantageous by allowing for fast analysis time, which is due in part to the enhanced mass transport effect [113,114]. Additionally, since cells are confined to nL-pL volumes within microchannels, the concentration of analyte does not become too dilute for detection. For this reason, MFSs have been used to study a variety of cellular responses, including cell differentiation [115], chemotaxis [116], and gene expression [117]. The integration of nanotechnology and cellular studies using a MFS has unarguably facilitated many possibilities in which nanotechnology can be applied to cellular studies.

Development strategies to deliver QDs intracellularly to their biological targets (*i.e.*, mitochondria, endoplasmic reticulum, and nucleus) with high efficiency and selectively enables QDs to serve as labels for monitoring intracellular protein trafficking in terms of their spatial and temporal activity as well as other QD-cellular applications such as imaging and *in vivo* sensing. Effective delivery and targeting is critical to better understand the many complex intracellular signaling and gene regulation pathways. Such analyses will ultimately provide much insight into the development of therapeutics that can efficiently target and modulate intracellular pathways for disease prevention [118]. One of the many challenges which preclude this goal is an optimal method for the delivery of QDs into cells [119,120]. Ideal characteristics of a delivery method should include: limited incubation time of cells with QDs in order to minimize cytotoxicity, reduction of the perturbation of cellular function, and prevention of the accumulation of introduced QDs into one specific organelle.

It has been proven that the ultimate fate of QDs within a cell is a function of the delivery method and the QD surface chemistry. The choice of introduction method will in part be a function of the desired application for the intracellular QDs. Targeted delivery of QDs will require a combination of facilitated and active delivery methods which direct QDs to a particular organelle and bypass the endosomal system of the cells. If only intracellular labeling is required, then passive and facilitated methods can be used for delivery. However, diagnostic applications and intracellular monitoring will require that the monodispersity of QDs be maintained, therefore, microinjection may be a more suitable option. Microfluidics clearly provides a state-of-the-art technology for the handling of QDs and cells, where multiple functions (*i.e.*, surface modification of QDs with nucleic acids, antibodies or peptides for facilitated delivery or intracellular sensing, electroporation of cells with QDs, cell sorting, dielectrophoretic separation of live and dead cells, and cell lysis) can be integrated within a single chip to provide a POC biosensor system.

**Figure 6.**
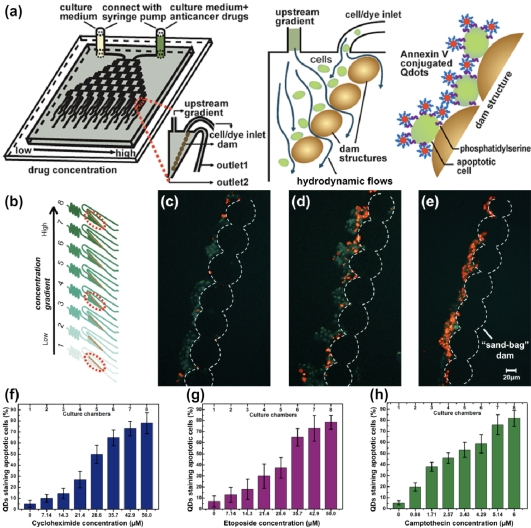
Schematic illustration of (**a**) a MFS with an integrated gradient generator and cell trapping sand-bag dam structures, depicting the detection of apoptotic cells trapped on the dam structures using Annexin V conjugated QDs; and (**b**) the eight chambers located within the MFS, each containing a parallel sand-bag dam structure for trapping the non-adherent cells. Chambers 1, 4, and 8 are illustrated in images c, d, and e, respectively; (**c**–**e**) Composite images show trapped human promyelocytic leukemic HL-60 cells stained by QDs (cells displaying red fluorescence are representative of apoptosis) treated with varying concentrations of cycloheximide: 0, 28.5, and 50.0 μM, respectively; (**f**–**h**) Correlative analysis of cells incubated in the presence of cycloheximide, etoposide, and camptothecin gradients, respectively, shows that the percentage of QDs stained HL-60 cells was dose-dependent. Reproduced with permission of the American Chemical Society [121].

The study conducted by Zhao *et al.* used QDs conjugated with Annexin V—a cellular protein that binds with the phosphatidylserine moieties in the plasma membrane of apoptotic cells—to screen for anti-cancer drugs. A MFS was implemented to analyze cell apoptosis at a single cell level (Figure 6) [121]. Analysis of apoptosis in microfluidics offers advantages such as real-time monitoring, which assists in the evaluation of the effect of cell heterogeneity on cell apoptosis, and provides rapid transduction by reducing the diffusion time of QD-Annexin V conjugates. Ultimately, these capabilities are unmatched by the conventional techniques used for studying cell apoptosis. The MFS used by Zhao and co-workers to study cell apoptosis incorporated a concentration gradient generator reported by Dertinger *et al.* [122]. It consisted of nodes and serpentine-like channels designed to split the initial stream of fluid into multiple channels, where a dilution step at the branch point was used to manipulate the composition of the starting solution at the inlet reservoir. The concentration gradient generator allowed for the interrogation of anti-cancer drug potency at various levels. In addition, the microfluidic network incorporated microstructures consisting of “sand-bag” dam structures to dock single cells [123,124] by applying an appropriate lateral pressure in the cell culture chambers. The effect of three anti-cancer drugs (e.g., cycloheximide, etoposide, and camptothecin) on cell apoptosis was considered in this study (Figure 6). The percentage of cells showing apoptosis were found to be proportional to the concentration and incubation time with the particular anti-cancer drug. In addition, it was found that camptothecin was the most potent of the three drugs investigated in terms of inducing cell apoptosis.

Zhang and co-workers performed fluorescence *in situ* hybridization (FISH) and QD-labeled immunofluorescent assay for the rapid detection of viable and non-viable microbial cells in a microfluidic device [125]. The device structure incorporated a filtering chamber consisting of trapping pillars separated by a distance of 1–2 micrometers for the isolation and concentration of microbial cells (*Giardia lamblia*). The advantage of performing an immunofluorescent assay in a microfluidic environment is the speed of analysis (*i.e.*, within minutes) due to enhanced mass transport, where continuous-flow of fluids through the channels facilitate otherwise time consuming washing steps.

Also, the consumption of minimal amounts of reagent and an increased signal-to-noise ratio were additional advantages of the assays conducted in a microfluidic channel, which provided a 1.5–5.7 fold higher signal-to-noise ratio for diluted (10 times) samples, as compared to that of assays done in bulk solution [125].

Morarka *et al.* developed a microfluidic-based immunosensing platform for the rapid detection of *Escherichia coli* (*E. coli*) using QD-antibody (anti-*E. coli*) conjugates as labels [126]. *E. coli* cells were adsorbed to the surface of the plasma oxidized PDMS microfluidic walls followed by the injection of QD-antibody conjugates through use of capillary action. The fluorescence intensity from the QD-antibody conjugates was found to be proportional to the concentration of cells in the sample. The drawback of this immunosensing approach was that the antigens (*i.e.*, *E. coli* cells) were captured along the length of the microfluidic channel by non-specific means, hence, requiring long incubation times for capture by antibody. Additionally, the reproducibility of the signal response was expected to be poor due to the continual washing of cells and antigen-antibody conjugates by continuous-flow.

In order to identify the potential of QDs as suitable substrates for *in vivo* applications, it is important to evaluate the factors that govern the cytotoxicity of QDs. Such studies will help elucidate and provide helpful insight into the criteria needed for consideration in the design of QD-based intracellular probes. Mahto *et al.* investigated the cytotoxic effects of MPA and cetyltrimethylammonium bromide/trioctylphosphine oxide (CTAB/TOPO) functionalized QDs on neuron-like PC12 cells using a compartmentalized microfluidic device consisting of two straight channels connected by microgrooves [127]. This channel network provided for the isolation of axons from their cell body, enabling localized exposure of QDs to axons and the cell body. The results suggested that the stability of a ligand on the surface of a QD plays an important role in maintaining cell viability. Concentrations as low as 0.427 nM of CTAB/TOPO-QDs showed significant signs of cytotoxicity within 2 h, directly causing morphological changes, cell shrinkage, degenerated axons, and loss of cell adhesion. On the contrary, 1.0 nM concentration of MPA-QDs showed no signs of cytotoxicity even after 6 h of exposure. Also, only the CTAB/TOPO-QDs showed signs of axonal degeneration caused by the potential involvement of reactive oxygen species upon preferential exposure to axons, while the cell body was found to be healthy. Mahto *et al.* also investigated the cytotoxic effects of CTAB/TOPO modified CdSe/ZnSe QDs on BALB/3T3 fibroblast cells studied under flow exposure conditions using a microfluidic device and compared the results with static exposure conditions using a well-plate system [128]. The microfluidic network used for the flow exposure conditions was based on two inlet reservoirs that merged into one main channel, where the main channel subsequently diverged into ten parallel channels. It was suggested that flow exposure based cytotoxicity studies using microfluidic technologies was a better platform for studying nanoparticle-based cell cytotoxicity because the continuous-flow technique could prevent the gravitational settling and aggregation of nanoparticles. Thus, a homogeneous distribution of nanoparticles could be maintained during exposure time. The problem with aggregation and settling of nanoparticles is that it can change the desired nanoparticle concentration, hence misrepresenting cytotoxicity. It has been reported in the literature that the aggregation of QDs might somehow be related to the cytotoxic effects of QDs [129]. Continuous-flow and perfusion of nutrients represent the physiological environment of the cell due to the presence of a circulatory system. This mediates entry of fresh cell culture medium while enabling the removal of cellular waste and ensuring a constant temperature and pH. Although the morphological changes due to QD cytotoxicity were found to be similar under flow and static conditions, cytotoxicity leading to apoptosis and deformed cells were found to be more pronounced under static conditions (30% cell survival) than compared to that under flow conditions (70% cell survival). The difference in cell survival was attributed to the additional physiological stress imposed on cells due to the settling of QDs on the surface of the cells. Ultimately, the leading cause for cell cytotoxicity was attributed to the release of Cd^2+^ ions from the QD core and the generation of reactive oxygen species inside the cells [129,130].

Investigation of cytotoxicity conducted under a continuous-flow scheme using microfluidics clearly demonstrated that the flow exposure conditions significantly affected cell viability. Thus, it is important that cytotoxicity studies conducted under static conditions be re-evaluated in terms of how the gravitational sedimentation of QDs changes the localized environment of the cell and the relevant implications on cell viability.

## 7. Summary and Outlook

This review highlights recent developments in the assimilation of QDs and microfluidic technologies onto a single platform to expand the available repertoire of biosensing and bioassay applications and to improve assay performance. A variety of QD-based biosensor platforms have emerged in recent years that consistently aim at developing systems that provide integrated, on-chip analysis systems capable of a wide variety of applications, essentially combining the advantages of both nano- and micro-technologies. QDs can be integrated into a MFS on an individual basis or by inclusion within other matrices, thereby ensuring immobilization or capture of biomolecular recognition elements, which is the basic operating principal for biosensing and bioassay systems.

It should be understood that the integration of microfluidics for biosensing remains an active area of scientific research. Several technological components and even some fundamental concepts are just now being developed, prompting continual innovation. Currently, the use of continuous-flow microfluidics dominates this developmental stage, and a large majority of available MFSs highlight the advantages achieved by implementing this type of system when compared to conventional biosensing techniques. The use of droplet-based microfluidics for biosensing applications is not well developed, and that of digital microfluidics is virtually nonexistent, suggesting untapped opportunities for further development of biosensor platforms. In short, the outlook is promising, and the synergistic use of QDs and microfluidics will offer many possibilities.

## Figures and Tables

**Table 1.** List of three different platforms in the field of microfluidics, including their operating principle, flow actuation, advantages, disadvantages, and applications.

	**Continuous-Flow Microfluidics**	**Droplet-Based Microfluidics**	**Digital Microfluidics**
**Operating Principle**	Use of micron sized channels to manipulate fluids in series	Emulsion droplets generated by capillary instability [28] between immiscible phases and confined within a microfluidic channel [29,30]	Manipulation of droplets using a patterned array of electrodes [31]
**Flow Actuation**	Pressure driven or electrokinetic flow	Pressure driven flow	External electric field
**Advantages**	Suitable for performing separations; surface can be chemically modified for grafting a biomolecular recognition element and control surface properties for the manipulation of electrokinetic flows, suitable for biosensing purposes	Each droplet can serve as a microreactor to perform synthesis and study reaction kinetics, thus providing an opportunity to confine reagents; avoid mixing and reagent cross contamination problems provided that mass transport between the droplet and external phase can be controlled	Ability to address each droplet individually [32], thus avoiding unwanted cross contamination between reagents; useful for tasks involving sequential treatment steps [33]
**Disadvantages**	Unwanted cross talk between different reagents is unavoidable; managing multiple reagents with precise control over their position and reaction time is complex	Unable to address each droplet individually as they are controlled in series [32]; encapsulated molecular species can partition themselves between two phases, thus not compatible with all the reagents as this will lead to sample losses; issues achieving a high degree of monodispersity	Not suitable for electrophoretic separations [34]; non-specific adsorption can cause complications [35]; device fabrication requires clean room facility; limited substrate choices, thereby limiting the choice of chemistries to modify the surface
**Applications**	Dielectrophoretic separation of cells [36], separations involving capillary zone electrophoresis (CZE), capillary gel electrophoresis (CGE), polymerase chain reaction (PCR), and DNA analyses [37]	Studies of reaction kinetics [38], electroporation of cells [39], biological assays [40,41]	Organic synthesis [42], biological assays [43], sample manipulation associated with proteomics research (*i.e.*, shotgun proteomics) [44]

**Table 2.** Examples of in-channel and microbead immobilization of QDs.

**Immobilization Method**	**Solid-phase Support**	
	***In-channel***	***Microsphere Bead***
Layer-by-layer assembly	[83]	[88]
Coordinating ligands	[84,85]	-
Physical entrapment	[86]	[89,90]
Avidin-biotin	[87]	[91,92]
Biomolecule tethering	[21]	-
Passive adsorption	-	[93]
